# Male incontinence: Pathophysiology and management

**DOI:** 10.4103/0970-1591.32071

**Published:** 2007

**Authors:** Rob Pickard

**Affiliations:** Newcastle University and Freeman Hospital, Newcastle Upon Tyne, UK. E-mail: r.s.pickard@ncl.ac.uk

The predominant cause of urodynamic stress incontinence in men is radical prostatectomy (RPP). Although the risk may be low in expert hands, the high number of procedures performed means that the complication is frequently encountered by urologists who need sufficient knowledge to explain the problem and outline alternatives in management as documented in the present review.[1]

The first difficulty we face is in defining the problem. Although any leakage is difficult to tolerate most men will accept the use of a few pads a day as a small price to pay for cancer cure. In contrast the need for condom sheath drainage signifies a severe problem which most men would want improved. In between will be many men who although having similar degrees of leak will vary in terms of how much bother they find it. To fix the problem we need to consider primary prevention, secondary prevention and finally treatment. Primary prevention in this circumstance means stopping doing RPP. This is potentially achievable for over 50% of men discovered to have localised prostate cancer who either will not die of their disease if left untreated or whose disease is too aggressive to be cured by local therapy.[2] Such disease stratification will have to await the development of biomarkers with validated predictive accuracy. Secondary prevention involves risk reduction by either pre-operative measures to strengthen the sphincter or refinement of operative technique to reduce sphincter damage.[3]

As far as treatment of an established problem goes we know that a period of ‘watchful waiting’ is essential since continence will improve during the 18 months after RPP. After this we lack any scientific evidence base to guide our advice to patients and therefore must rely on common sense and expert opinion. For men with mild incontinence it seems sensible to advise continued conservative measures such as pelvic floor exercise and duloxetine. For the severe group requiring a sheath or catheter, the artificial urinary sphincter remains the standard, albeit costly, option. It is those with moderate leakage who provide the impetus to develop other management options such as ventral urethral compression by slings. The use of such devices is not new, but the prosthetic materials are. The monofilament and macroporous design of modern tapes seem well suited for incorporation into tissue with reduction in risk of infection and erosion which complicated older procedures. There are a number of uncontrolled phase II studies supported by industry that suggest reasonable short-term efficacy in men with mild or moderate incontinence following RPP but what we need next are higher quality randomised studies that include the patient's viewpoint and cost-effectiveness analysis.[4]

In summary tackling the problem by refining the indication for RPP, improving operative technique and validating less costly methods of treatment appears the way forward.
